# Reduced autoimmunity associated with deletion of host CD73

**DOI:** 10.1093/immhor/vlae004

**Published:** 2025-01-23

**Authors:** Beanna Okeugo, Shabba A Armbrister, Rhea C Daniel, Zeina M Saleh, Jessica Wang, Salomea Giorgberidze, J Marc Rhoads, Yuying Liu

**Affiliations:** Department of Pediatrics, Division of Gastroenterology, McGovern Medical School, The University of Texas Health Science Center at Houston, Houston, TX, United States; Department of Pediatrics, Division of Gastroenterology, McGovern Medical School, The University of Texas Health Science Center at Houston, Houston, TX, United States; Department of Pediatrics, Division of Gastroenterology, McGovern Medical School, The University of Texas Health Science Center at Houston, Houston, TX, United States; Department of Pediatrics, Division of Gastroenterology, McGovern Medical School, The University of Texas Health Science Center at Houston, Houston, TX, United States; Department of Pediatrics, Division of Gastroenterology, McGovern Medical School, The University of Texas Health Science Center at Houston, Houston, TX, United States; Department of Pediatrics, Division of Gastroenterology, McGovern Medical School, The University of Texas Health Science Center at Houston, Houston, TX, United States; Department of Pediatrics, Division of Gastroenterology, McGovern Medical School, The University of Texas Health Science Center at Houston, Houston, TX, United States; Department of Pediatrics, Division of Gastroenterology, McGovern Medical School, The University of Texas Health Science Center at Houston, Houston, TX, United States

**Keywords:** dermatitis, IPEX syndrome, primary immune deficiency, regulatory T cell, scurfy, thymocytes

## Abstract

CD73 is ubiquitously expressed and regulates critical functions across multiple organ systems. The sequential actions of CD39 and CD73 accomplish the conversion of adenosine triphosphate to adenosine and shift the adenosine triphosphate–driven proinflammatory immune cell milieu toward an anti-inflammatory state. This immunological switch is a major mechanism by which regulatory T (Treg) cells control inflammation. Foxp3 engages in Treg development and function. Foxp3 mutations result in the scurfy (SF) mouse phenotype and a rapidly lethal lymphoproliferative syndrome. We generated double knockout (KO) mouse (CD73KOSF) by breeding heterozygous Foxp3sf/J females to CD73KO male mice to remove host CD73. We initially aimed to use these mice to identify a specific probiotic-CD73 effect, previously shown for *Limosilactobacillus reuteri* DSM 17938. We expected CD73 deletion to enhance the severity of autoimmunity in SF mice. However, we unexpectedly observed that KO of host CD73 in SF mice clinically reduced the severity of autoimmunity including reduced ear thickness, increased ear size, and less deformed ears, along with less dry and brittle skin. KO of CD73 in SF mice significantly reduced the numbers of CD4^+^ and CD8^+^T cells in spleen and blood. We identified that KO of CD73 in SF mice reduced the numbers of T cells in the thymus compared with those in SF mice, indicating that the milder clinical phenotype may be due to reduced central and peripheral lymphoproliferation. These new findings suggest targeting CD73 could improve T cell–mediated dermatitis, one of the most common symptoms in Treg deficiency–associated primary immune deficiencies.

## Introduction

Regulatory T (Treg) cells are crucial for maintaining central and peripheral tolerance.^1^ The Forkhead box protein 3 (Foxp3) gene, a master transcription factor of Treg cells, is involved in Treg development, stability, and function.^2^ Foxp3^+^Treg cells suppress conventional T cells and are essential for immunological tolerance. Foxp3 mutations result in the scurfy (SF) mouse phenotype, exhibiting a severe autoimmune phenotype with multiorgan failure and early death due to a lymphoproliferative syndrome. The SF mouse models a human monogenic primary immune deficiency characterized by immune dysregulation, polyendocrinopathy, enteropathy and X-linked inheritance (IPEX syndrome),^3^^,^^4^ with clinical hallmarks including eczema, enteropathy, type 1 diabetes, thyroiditis, hemolytic anemia, and thrombocytopenia.^3^^,^^4^

One of the mechanisms by which Treg cells control inflammatory T cells is via a coupling of cell surface ectoenzymes named CD39 and CD73, which convert extracellular adenosine triphosphate (ATP), a potent proinflammatory mediator, into its anti-inflammatory product adenosine.^1^^,^^5^ Adenosine interacts with adenosine receptor 2A (A_2A_) expressed on T cells to intervene with immunosuppressive and anti-inflammatory activities.^1^^,^^5^^,^^6^ Therefore, in SF mice, inflammatory T helper 1 (Th1) and Th2 cells and their associated cytokines in the spleen and plasma are significantly increased due to loss of control by Treg cells and, at least in part, by loss of Treg-mediated CD39/CD73–generated adenosine.^7^ We demonstrated previously that dynamic changes in autoimmunity and gut microbial dysbiosis evolved rapidly during the short lifespan of SF mice.^7^ We were able to modify these changes by intragastric administration of the probiotic *Limosilactobacillus reuteri* DSM 17938, resulting in a marked prolongation of lifespan from <1 month to >4 months.^7^ We also discovered that this probiotic which encodes ecto-5′-nucleotidase (CD73) activity, has protective effects against SF inflammation through probiotic-generated adenosine and the adenosine metabolite inosine.^6–9^ Herein, we generated SF mice with an additional knockout (KO) of host CD73 (CD73KOSF), expecting to enhance the severity of the clinical phenotype in SF mice to be used as a tool to evaluate probiotic-CD73 activity. We now report an unexpected finding that the KO of CD73 improved clinical severity, especially reducing autoimmune dermatitis, in this disease model.

## Materials and methods

### Mice

**Figure 1.**
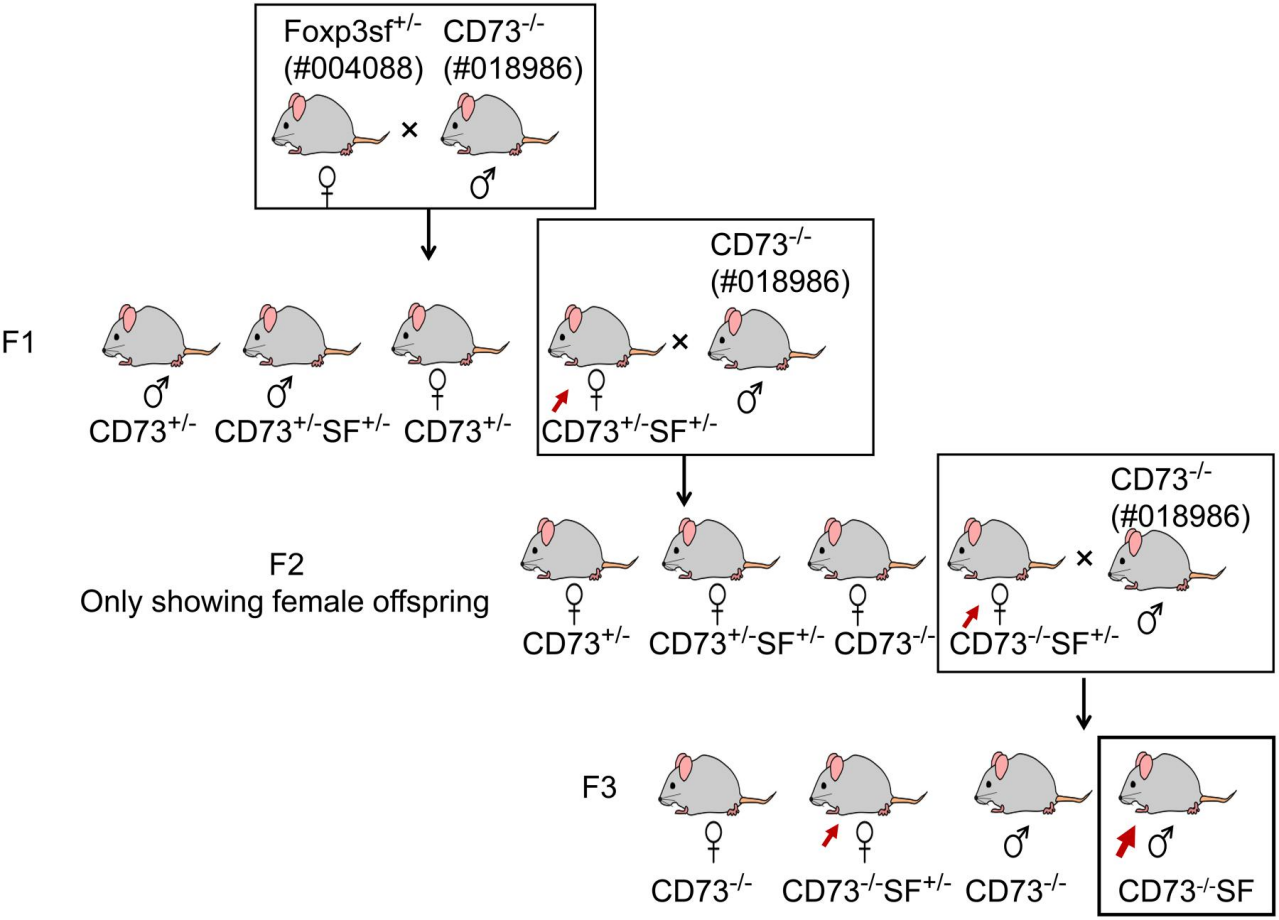
Generation of CD73KOSF mice: core genotype and breeding scheme. Red arrows indicate the confirmed mouse genotype used for setting up breeding pairs and CD73KOSF mice for the experiments.

C57BL/6J WT mice (000664), heterozygous B6.Cg-Foxp3sf/J **(**004088) female mice, and B6.129S1-Nt5e^tm1Lft^/J (018986, CD73KO) mice, 6 to 8 wk old, were purchased from the Jackson Laboratory and were acclimatized for 2 wk before setting up breeding pairs for generating wild-type (WT), SF (B6.Cg-Foxp3sf/Y), CD73KO, and CD73KOSF mice used for the experiment. SF mice were generated by breeding C57BL/6J WT male mice to heterozygous B6.Cg-Foxp3sf/J female mice. To generate CD73KOSF (Fig. 1), the first step was breeding CD73KO male mice with heterozygous SF female mice to collect heterozygous CD73 heterozygous SF (CD73hetSFhet) female mice; the second step was breeding CD73hetSFhet female mice with CD73KO male mice to collect CD73KOSFhet females; and the last step was breeding CD73KOSFhet female mice with CD73KO male mice to generate CD73KOSF mice; mouse strains were confirmed by genotyping at each step. Because the Foxp3 gene is located on the X chromosome, only males had SF features, with a 25% probability of total offspring from each litter being SF or CD73KOSF mice. Therefore, SF or CD73KOSF mice were collected from at least 3 different cages and only male mice were used in this study. The mice were housed under a 12-h light/dark cycle and temperatures of 18 °C to 23 °C with 40% to 60% humidity. They had access to food and water ad libitum in a specific pathogen–free animal facility at the University of Texas Health Science Center at Houston (UTHealth). The study was carried out in accordance with the Recommendations of the Guide for the Care and Use of Laboratory Animals of the National Institutes of Health. The Institutional Animal Care and Use Committee of UTHealth approved the study (protocol numbers: AWC-20-0032 and AWC-22-0112).

### Evaluation of mouse clinical phenotype, pathology, and histopathology

SF mice generally developed the SF phenotype at 13 days of life (DOL), and early deaths were noted around 24 to 28 DOL.^7^ In this study, tissues were collected from mice at the weaning date (day 21 [d21] of age) for analysis. In a separate set of experiments, mice were observed after weaning at d21 and the date of death was recorded for each mouse to calculate the survival rate for each study group.

The numbers of mice in each group were the following: WT, n = 8; SF, n = 7; CD73KOSF, n = 7; and CD73KO, n = 9 for d21 analysis and WT, n = 10; SF, n = 17; CD73KOSF, n = 19; and CD73KO, n = 10 for survival analysis.

Each mouse was weighed and photographed at d21 before euthanization for tissue collection. SF mice demonstrated deformed ears compared with normal mice. The areas of ears were measured by using ImageJ (FIJI) (National Institutes of Health morphometry software. SF tail dermatitis based on eye observation had 3 categories: normal (no dermatitis), mild (with patchy or less than half of the total tail having dermatitis or small patchy necrosis, no swelling), or severe (greater than or equal to half of the total tail having dermatitis, swelling, bleeding, or skin necrosis). Mouse tissue including ear, tail, thymus, liver, and lung samples from each mouse were collected fixed with formalin, processed, and stained with hematoxylin and eosin (H&E); in addition, immunohistochemical staining of CD3^+^T cells on mouse thymus sections was performed with a Dako Omnis system (Agilent) and Agilent CD3 polyclonal antibody with high pH antigen retrieval by Histology Laboratory, Department of Pathology and Laboratory Medicine at UTHealth. Tail epidermis + dermis thickness and the area of lymphocyte infiltration in the liver and lung from hematoxylin and eosin sections were measured using ImageJ. Under the designated magnification, at least 5 microscopic fields per mouse sample were measured and mean ± SD in pixels were calculated to compare among the groups.

### In vitro tissue preparation and staining cells for flow cytometry analysis

Single-cell suspensions from spleen and thymus were obtained by gently fragmenting and filtering the tissues through 40-µm BD Falcon Cell Strainers into RPMI 1640 (Sigma-Aldrich) complete medium. MACS buffer consisting of phosphate-buffered saline, 0.5% bovine serum albumin (HyClone; GE Healthcare Life Sciences), and 2 mM EDTA (Lonza) were used for washing the cells. Finally, cells were resuspended in MACS buffer for surface and intracellular staining using specific anti-mouse antibodies. The antibodies that we used were CD4 (GK1.5) conjugated with peridinin-chlorophyll protein/cyanine 5.5 (PerCP/Cy5.5), CD8a (53-6.7) conjugated with brilliant violet 421 (BV421), CD39 (Duha59) conjugated with phycoerythrin, CD73 (TY/11.8) conjugated with allophycocyanin, and Foxp3 (FJK-16s) conjugated with Alexa Fluor 488. For intracellular Foxp3 staining, the eBioscience Foxp3/Transcription Factor Staining Buffer Set was used according to the manufacturer’s instructions to permeabilize cells. All antibodies were purchased from BioLegend. The data from all samples were acquired on BD LSRFortessa (BD Biosciences) and analyzed using FlowJo software version 10.8.1 (BD).

### Plasma cytokine and chemokine assay

Blood from each mouse was collected and plasma was isolated and stored immediately at −80 °C after the mice were euthanized at d21. Plasma cytokine levels including interferon-γ (IFN-γ), interleukin (IL)-2, IL-4, IL-5, IL-6, IL-10, IL12p70, IL-15, IL-27/p28/IL-30, IL-33, tumor necrosis factor-α (TNF-α), and chemokine levels including macrophage inflammatory protein-1 alpha (MIP-1α), MIP-2, monocyte chemoattractant protein 1 (MCP-1), and IFN-γ-induced protein 10 (IP-10) were assessed using mouse multiplex proinflammatory panel and chemokine panel kits from Meso Scale Discovery, according to the manufacturer’s protocols. Signals were captured and calculated by Imager 2400 (Meso Scale Discovery). The levels of cytokines and chemokines were expressed in pg/mL.

### Statistical analysis

Statistical significance was determined by 1-way analysis of variance corrected for multiple comparisons with Tukey’s post-test, or 2-way analysis of variance for multiple comparisons with a Bonferroni test. Fisher’s exact test for contingency tests was performed to compare the incidences of mice with mild scurfy tail and mice with ≥ 30 or <30  DOL. Kaplan-Meier survival curves were graphed and the comparison was analyzed using Log-rank test with chi-square test. The statistical analysis was performed by using GraphPad Prism version 9.4.1 (GraphPad Software). Data are presented as mean ± SD. *P* values <0.05 were considered statistically significant.

## Results

### Reduced SF clinical phenotype with KO of host CD73 in SF mice

**Figure 2.**
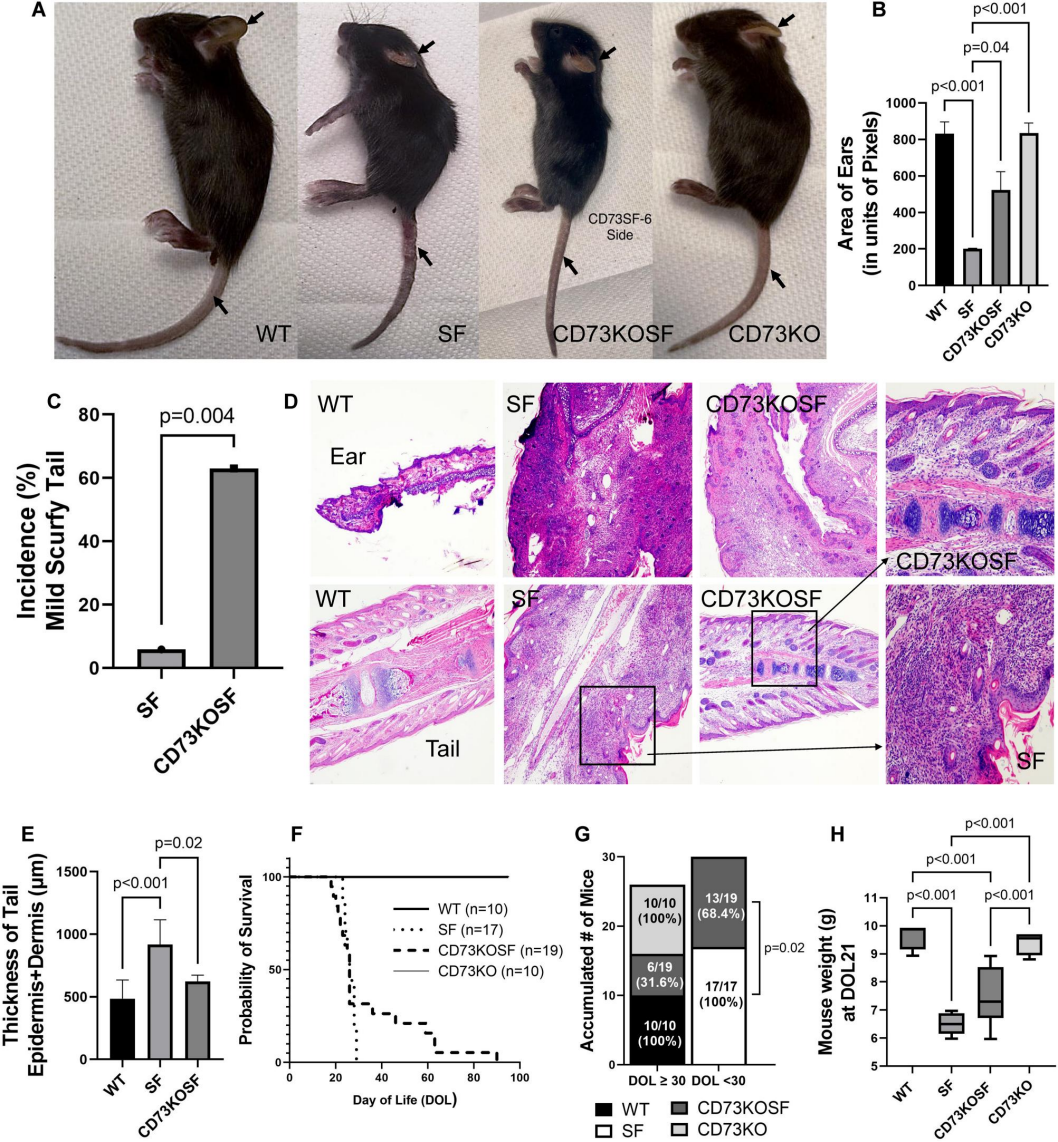
Evaluation of mouse clinical phenotype. (A) phenotypic observations of ears and tails of mice compared among the different genotype mouse groups including WT, SF, CD73KOSF, and CD73KO. (B) Area of ears measured by using ImageJ software based on each mouse photograph. Values represent mean ± SD, and 1-way analysis of variance multiple comparisons with Tukey’s post-test. (C) Incidence of mild SF tail, comparing CD73KOSF vs. SF; note that the definition of normal, mild, and severe has been described in the Materials and Methods. Fisher’s exact test was used to test the differences. (D) H&E staining histological observations of ears and tails of mice, 40×magnification; square areas show 200× magnification for CD73KOSF and SF, as indicated by arrows. (E) Tail epidermis + dermis thickness was measured by using ImageJ software based on H&E staining histology at 40× magnification. (F) Kaplan-Meier survival curves for each group of mice. (G) Accumulated number of mice divided by DOL ≥30 and DOL < 30; Fisher’s exact test was used. (H) Mouse weight (g) at d21 of age. Values represent mean ± SD, 1-way analysis of variance multiple comparisons with Tukey’s post-test. For d21 analysis, WT n = 8, SF n = 7, CD73KOSF n = 7, and CD73KO n = 9. For survival analysis, WT n = 10, SF n = 17, CD73KOSF n = 19, CD73KO n = 10. Significant *P* values (*P *< 0.05) are indicated in the graphs.

High levels of CD73 (ecto-5′-nucleotidase) have been implicated in immune suppression.^10–12^ Therefore, in SF mice, we expected that KO of host CD73 would enhance disease progression and severity. Unexpectedly, we observed that CD73KOSF mice demonstrated milder clinical symptoms than SF mice. The SF mice had thicker and more deformed ears, with a hunched position, and the tails showed visible skin dermatitis and necrosis when compared with normal WT mice. However, each of the CD73KOSF mice showed intermediate severity, with small, rounded, and thickened ears without deformity (Fig. 2A). We measured the area of 1 ear of each mouse from photographs by using ImageJ software to survey the difference of the size of ears and compare the different genotypes. The mean surface area of ears in CD73KOSF mice was significantly increased compared with that in SF mice (*P *= 0.04) (Fig. 2B). The tails of CD73KOSF mice also showed less thickening, dermatitis and necrosis compared with SF mice (Fig. 2A). We arbitrarily defined the severity of the tail dermatitis based on pathological evaluation of the affected area of dermatitis and necrosis, which was graded as normal, mild, or severe. We found that, for most of the CD73KOSF mice, the tails showed patchy inflammation, with less than half of the area of a tail showing dermatitis or small patchy necrosis; none were swollen and some of the tails looked normal. The incidence of mild scurfy tail dermatitis in CD73KOSF mice was 63%, compared with that in SF mice (6%) (*P *= 0.004) (Fig. 2C). Both ear and tail histology revealed inflammation with leukocyte infiltration in SF mice; however, leukocyte infiltration in CD73KOSF mice was reduced (Fig. 2D). We measured the combined thickness of epidermis and dermis in the tails and found that CD73KOSF mice had reduced tail thickness compared with SF mice (*P *= 0.02), whereas in SF mice, the tail thickness was significantly increased compared with WT mice (*P *< 0.001) (Fig. 2E).

We observed each mouse daily to see if CD73KOSF mice had a better survival rate than SF mice (Fig. 2F). We found that all SF mice (17 of 17 [100%]) died within 30  DOL; however, fewer, 13 (68.4%) of 19, CD73KOSF mice died within 30 DOL (*P *= 0.02). Of the CD73KOSF mice surviving > 30 DOL, the maximal survival was 90 DOL (Fig. 2F and G). The weights of SF mice at d21 of age (6.5 ± 0.4 g) were significantly reduced compared with those of WT mice (9.6 ± 0.5 g) and CD73KO mice (9.4 ± 0.4 g) (all *P *< 0.001). However, the weights of CD73KOSF mice (7.4 ± 1.0 g) at d21 were comparable to those of SF mice (*P *= 0.08) (Fig. 2H).

### KO of host CD73 and peripheral T lymphocyte proliferation in SF mice

**Figure 3.**
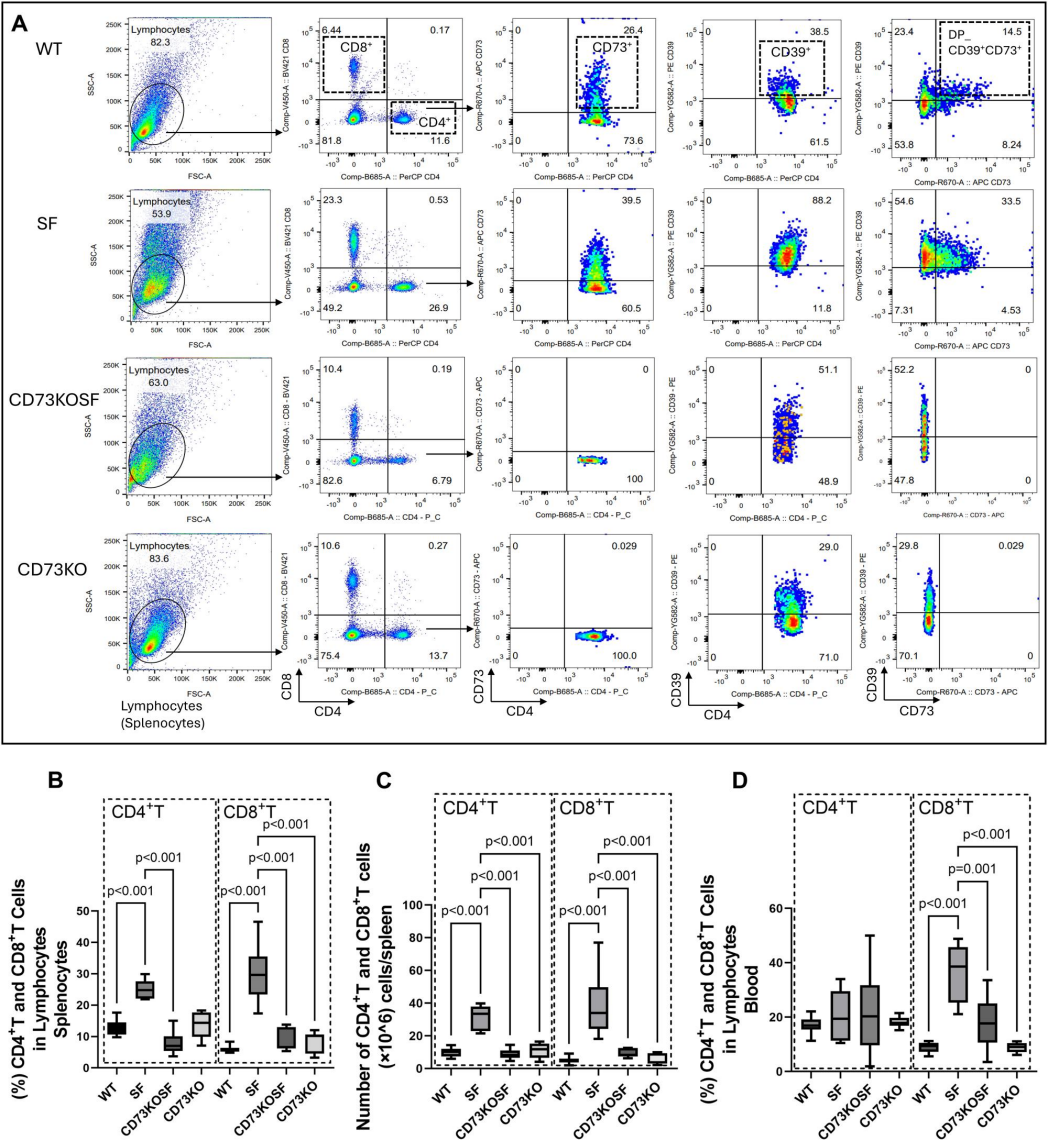
Flow cytometry analysis of CD4^+^T and CD8^+^T cells in the spleen and blood of mice. (A) Flow cytometry images showed the definition of CD4^+^ and CD8^+^T cells among lymphocytes, and CD73^+^, CD39^+^, and DP_CD39^+^CD73^+^ among CD4^+^T cells; the same analysis among CD8^+^T cells was performed (not shown). (B) % of CD4^+^ and CD8^+^T cells among lymphocytes in the splenocytes isolated from the mice. (C) the number of CD4^+^ and CD8^+^T cells (×10^6^) per spleen. (D) % of CD4^+^ and CD8^+^T cells in the blood of mice. Values represent mean ± SD, 2-way analysis of variance for multiple comparisons with a Bonferroni test. Significant *P* values (*P *< 0.05) are indicated in the figures. FSC-A = forward scatter area; SSC-A = side scatter area.

Due to Foxp3^+^Treg deficiency, SF mice exhibit multiorgan system inflammation and always die early. We previously reported that T cells including CD4^+^ and CD8^+^T cells are increased in the peripheral blood of SF mice.^13^ In the current study, we further analyzed the effect CD73 KO on CD4^+^ and CD8^+^T cells in the spleen and blood. We observed that KO of CD73 in SF mice reduced the % of cells in lymphocytes and the cell numbers of both CD4^+^T and CD8^+^T cells in the spleen (Fig. 3A–C) and reduced % of CD8^+^T cells in blood (Fig. 3D) compared with SF mice (all, *P *< 0.001).

### CD73 expression in non-Treg cells in SF mice, and effect of KO of CD73 in SF mice on CD39^+^T cells

CD73, together with CD39, is responsible for a cascade converting ATP to adenosine diphosphate, ultimately leading to the release of immunosuppressive adenosine in an inflammatory environment.^14^ CD73 and CD39 are expressed on the surface of Treg cells; and the CD73/CD39 pathway has been highlighted as one of mechanisms by which Treg cells control inflammatory T cells.^7^^,^^15^ We analyzed CD73 and CD39 expression on T cell surfaces including CD73^+^ among CD4^+^T cells or CD8^+^T cells, CD39^+^ among CD4^+^T cells or CD8^+^T cells, and double positive (DP) CD39^+^CD73^+^ among CD4^+^T cells or CD8^+^T cells (Fig. 3A shows representative flow diagrams in the CD4^+^T cell population) to identify the % of these T cells in Treg cell–deficient SF mice and to examine the CD39^+^T cell numbers when CD73 is knocked out.

**Figure 4.**
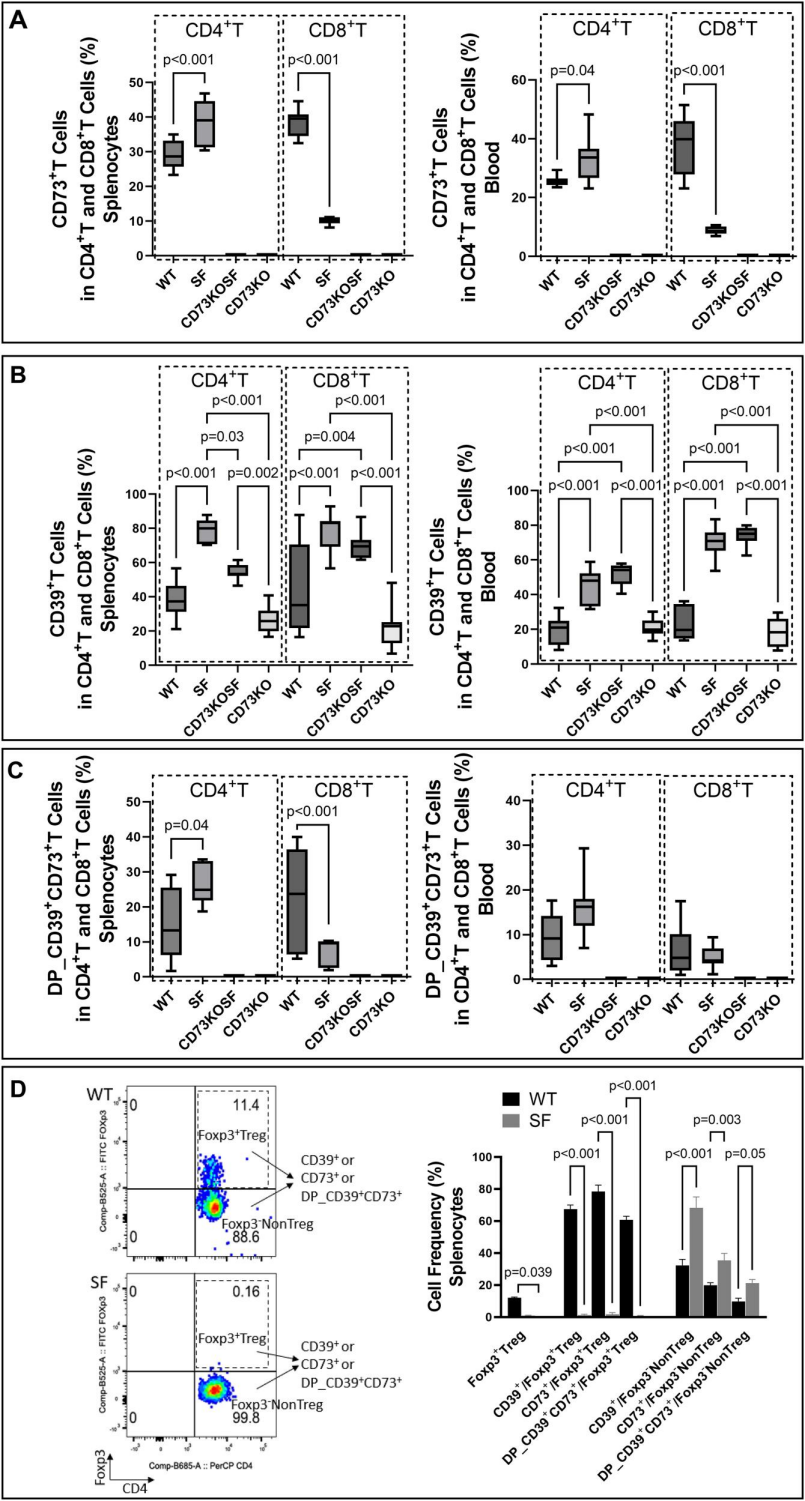
The percentage of CD39^+^ and CD73^+^T cells in the spleen and blood analyzed by flow cytometry compared with the groups of mice. (A) % of CD73^+^in CD4^+^T cells and % of CD73^+^in CD8^+^T cells in the spleen (left) and in the blood (right). (B) % of CD39^+^in CD4^+^T cells and % of CD39^+^in CD8^+^T cells in the spleen (left) and in the blood (right). (C) % of DP_CD39^+^CD73^+^in CD4^+^T cells and % of DP_CD39^+^CD73^+^in CD8^+^T cells in the spleen (left) and in the blood (right). (D) Flow cytometry images showed the definition of Foxp3^+^Treg and Foxp3^−^NonTreg among CD4^+^T cells of the splenocytes from WT (top) and SF (bottom) mice, and further identified the CD39^+^, CD73^+^, and DP_CD39^+^CD73^+^populations in Foxp3^+^Treg and Foxp3^−^NonTreg, respectively (left), and bar graphs showed % of CD39^+^, CD73^+^ and DP_CD39^+^CD73^+^ among Foxp3^+^Treg and Foxp3^−^NonTreg comparing between WT and SF mice (right). Values represent mean ± SD, and we used 2-way analysis of variance for multiple comparisons with a Bonferroni test. Significant *P* values (*P *< 0.05) are indicated in the figures.

Comparing SF mice with WT mice, we found that the % of CD73^+^CD4^+^T cells was increased, while the % of CD73^+^CD8^+^T cells was reduced in the spleen (Fig. 4A, left) and blood (Fig. 4A, right). We also confirmed absence of CD73^+^T cells in the spleen (Fig. 4A, left) or blood (Fig. 4A, right) of CD73KOSF and CD73KO mice. Among CD4^+^ or CD8^+^T cell populations used to define CD73^+^, CD39^+^, and DP_CD39^+^CD73^+^cells, we found that % of CD39^+^T cells (in both CD4^+^ and CD8^+^ T cells) were significantly increased in SF mice in the spleen (Fig. 4B, left) and in the blood (Fig. 4B, right) (all, *P *< 0.001) compared with WT mice. Knockout of CD73 in SF mice markedly reduced the CD39^+^CD4^+^ population in the spleen (*P *= 0.03) but did not alter CD39^+^CD4^+^T cells in the blood, or CD39^+^CD8^+^ T cells in the spleen and blood (Fig. 4B). In addition, KO of CD73 in SF mice significantly increased % of DP_CD39^+^CD73^+^ cells in CD4^+^T cell populations (*P *= 0.04), while we found reduced % of DP cells in CD8^+^T cell populations (*P *< 0.001) in the spleen of SF mice compared with WT mice (Fig. 4C, left). This was similarly affected as measured by the % of CD73^+^cells in CD4^+^T and CD8^+^T in the spleen of mice (Fig. 4A, left). Importantly, CD39^+^ and CD73^+^ T cells were numerous among Foxp3^+^Treg cells and Foxp3^−^NonTreg cells in the spleen (Fig. 4D, left), respectively. In fact, over 60% of Foxp3^+^Treg cells expressed CD39^+^ or CD73^+^ or DP-CD39^+^CD73^+^ in the spleen of WT mice (Fig. 4D, right). We noted that CD39 and CD73 are not only expressed on Foxp3^+^Treg cells but are also expressed on Foxp3^−^NonTreg cells in the splenocytes. About 30% of Foxp3^−^NonTreg cells expressed CD39, 20% of Foxp3^−^NonTreg cells expressed CD73, while 10% of Foxp3^−^NonTreg cells expressed both CD39 and CD73 in the spleen of WT mice. Interestingly, these cells were significantly increased in the spleen of SF mice compared with WT control mice (Fig. 4D, right).

### Host CD73 and maintenance of thymocytes in SF mice

**Figure 5.**
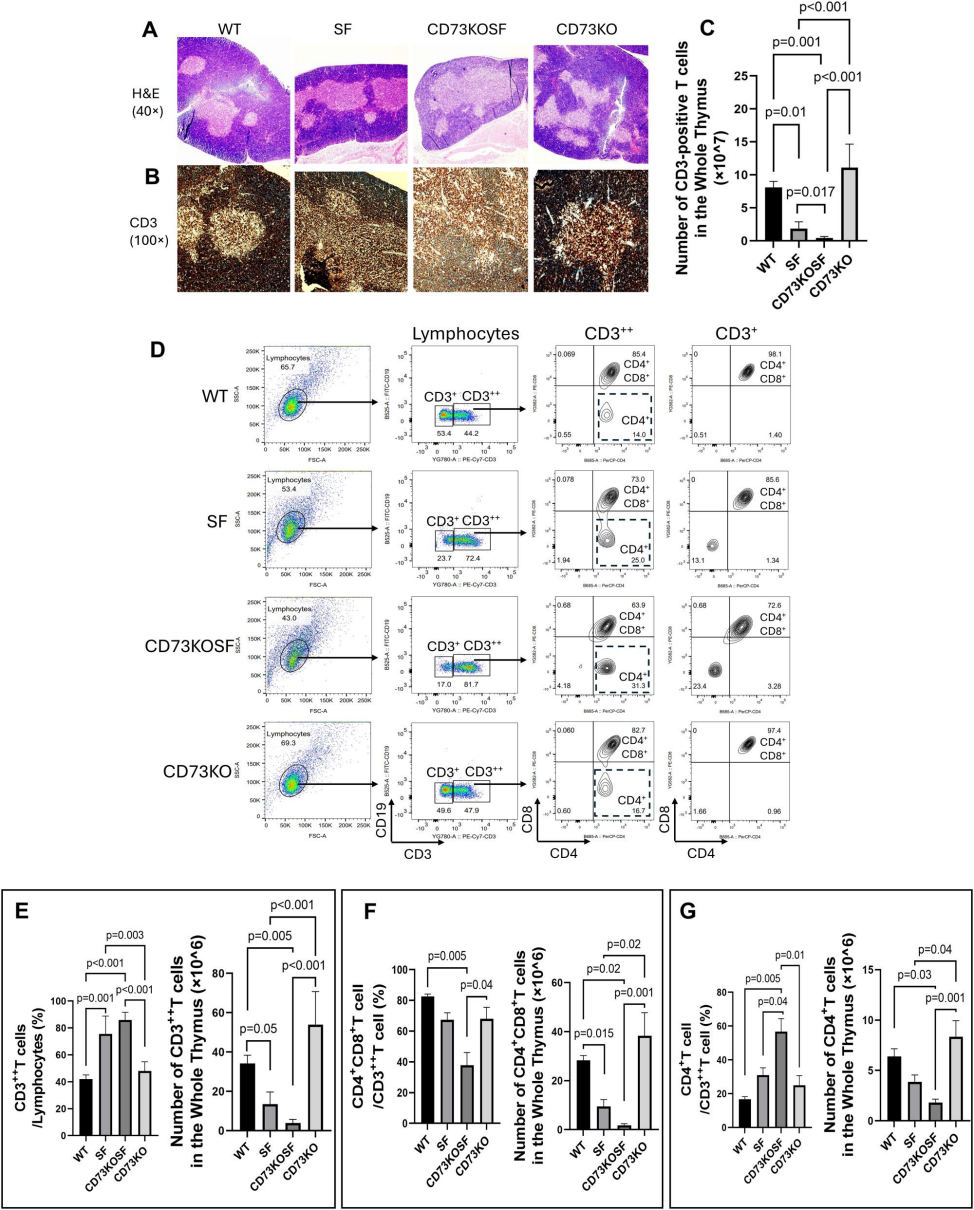
T cells in the thymus. (A, B) Thymus histology images with H&E staining (40×magnification) (A) and anti-CD3 staining (100×magnification) (B) showing T cells in the outer cortex area and inner medulla area of the thymus of each mouse in different groups. (C) The number of CD3-positive T cells in the whole thymus in each group by flow cytometry analysis. (D) Flow cytometry images on defining CD3^+^ and CD3^++^T cells among lymphocytes in the thymus; we further defined DP CD4^+^CD8^+^T cells and single CD4^+^T cells among the CD3^++^T and CD3^+^T populations, respectively. (E) % of CD3^++^T cells among lymphocytes (left) and the number of CD3^++^T cells in the whole thymus (×10^6^) (right) compared with the groups. (F) % of DP_CD4^+^CD8^+^T cells among the CD3^++^T cell population (left) and the number of DP_CD4^+^CD8^+^T cells in the whole thymus (×10^6^) (right) in the groups; and G. % of single CD4^+^T cells among CD3^++^T cell population (left) and the number of CD4^+^T cells in the whole thymus (×10^6^) (right) in the groups. Values represent mean ± SD. One-way analysis of variance multiple comparisons with Tukey’s post-test. Significant *P* values (*P *< 0.05) are indicated in the figures. FSC-A = forward scatter area; SSC-A = side scatter area.

Because we observed that KO of host CD73 in SF mice reverses severe CD4^+^T and CD8^+^T cells in the peripheral blood and spleen (Fig. 3A–D), we speculated that KO of CD73 may affect central T cell ontogenesis. The thymus plays a crucial role in the development of T lymphocytes by providing an inductive microenvironment to commit proliferation of progenitors, T cell receptor gene rearrangements, and the differentiation of thymocytes to T cells.^16^ Thymocytes are predominately (>80%) DP_CD4^+^CD8^+^T cells.^16^ In tissue histological analysis of the thymus, we observed by both H&E staining (Fig. 5A) and immunohistochemical CD3 staining (Fig. 5B) that the thymus of double KO mice was characterized by reduced lymphocytes and CD3^+^T cells in the cortex and medulla of the thymus. We further confirmed this finding by flow cytometry analysis and cell count, which showed that number of CD3-positive T cells in the whole thymus was significantly reduced in SF mice compared with WT mice (*P *= 0.01) and CD73KO mice (*P *< 0.001); the CD3-positive T cells in the thymus were further significantly reduced in CD73KOSF mice compared with SF mice (*P *= 0.017) (Fig. 5C). Among the CD3-positive T cell population, we observed a shift of the cells from lower expression of CD3 (CD3^+^) to higher expression of CD3 (CD3^++^) in SF mice and CD73KOSF mice but not in WT and CD73KO mice (Fig. 5D), with significantly increased % of CD3^++^T cells among lymphocytes in SF mice and CD73KOSF mice compared with WT mice or CD73KO mice (all *P *< 0.001) (Fig. 5E, left), however, the CD3^++^T cell numbers in the whole thymus were still reduced in SF mice and CD73KOSF mice compared with those in WT and CD73KO mice (Fig. 5E, right). In addition, among these CD3^++^T cell population, CD73KOSF mice showed significantly reduced both % and cell numbers of DP CD4^+^CD8^+^T cells (Fig. 5F) and cell numbers of single CD4^+^T cells (Fig. 5G, right), despite the presence of increased % of single CD4^+^T cells (Fig. 5G, left) compared with SF, WT, and CD73KO mice, respectively.

### Host CD73 deletion, histology, and severe systemic inflammation secondary to Treg cell deficiency in SF mice

**Figure 6.**
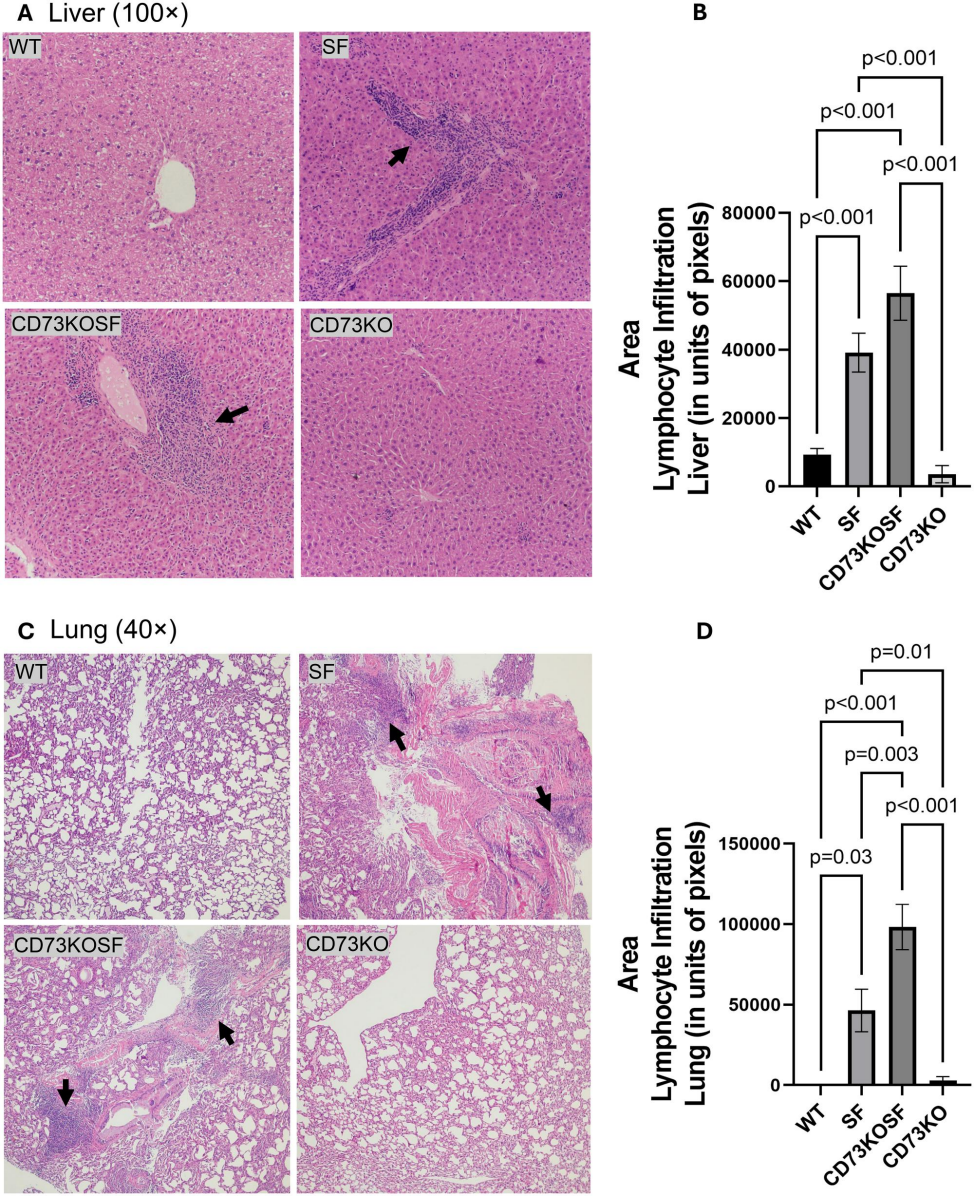
Liver and lung histological evaluation. (A) Representative histological images with H&E staining of livers (100× magnification; the arrows indicate lymphocyte infiltration) from different mice. (B) Area of lymphocyte infiltration in the liver measured by using ImageJ software under 40× magnification. (C) Representative histological images with H&E staining of lungs (40× magnification; the arrows indicate lymphocyte infiltration) from different mice. (D) Area of lymphocyte infiltration in the lung was measured by using ImageJ software under 40× magnification. Values represent mean ± SD, and significance was assessed using 1-way analysis of variance with multiple comparisons and Tukey’s post-test. Significant *P* values (*P *< 0.05) are indicated in the figures.

Because we observed that KO of CD73 in SF mice reduces the clinical SF phenotype, specifically the scaly dermatitis, we investigated whether KO of CD73 in SF mice could reduce SF-associated systemic inflammation including multiorgan inflammation and plasma cytokines. We evaluated the degree of inflammation in the liver (Fig. 6A and B) and the lung (Fig. 6C and D) by measuring the area of lymphocyte infiltration of H&E staining histological images. We found that CD73KOSF mice demonstrated severe liver and lung inflammation to a level comparable to that seen in SF mice.

**Figure 7.**
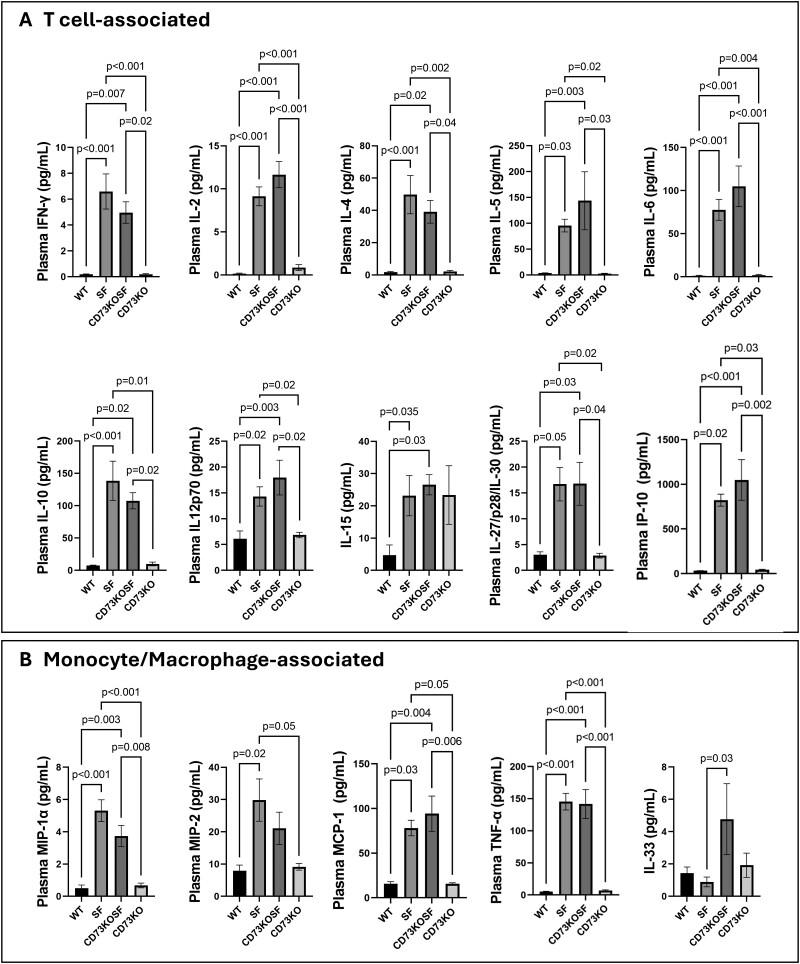
Plasma levels of cytokines and chemokines in the different groups of mice. (A) T cell–associated cytokines and chemokines: IFN-γ, IL-2, IL-4, IL-5, IL-6, IL-10, IL12p70, IL-15, IL-27/p28/IL-30, and IP-10. (B) Monocyte/macrophage-associated cytokines and chemokines: MIP-1α, MIP-2, MCP-1, TNF-α, and IL-33. Values represent mean ± SD; 1-way analysis of variance with Tukey’s post-test for multiple comparisons. Significant *P* values (*P *< 0.05) are indicated in the figures.

To confirm unchanged multisystem inflammation, we measured plasma cytokine levels. We found that levels of both T cell–associated cytokines (IFN-γ, IL-2, IL-4, IL5, IL-6, IL-10, IL12p70, IL-15, IL-27/p28/IL-30, and IP-10) (Fig. 7A) and monocyte/macrophage-associated cytokines/chemokines (MIP-1α, MIP-2, MCP-1, and TNF-α) (Fig. 7B) in the plasma of SF mice were significantly elevated compared with WT mice. Unlike skin inflammation, these cytokine/chemokine levels in the plasma of CD73KOSF mice were comparable to levels of SF mice, indicating that KO of host CD73 fails to rescue severe systemic inflammation caused by Treg-deficiency. Similarly, but uniquely, we found that monocyte/macrophage-associated IL-33 levels were only increased in CD73KOSF mice but not in SF mice (*P *= 0.03) (Fig. 7B).

## Discussion

In this study, we report an unexpected finding in the Foxp3^+^Treg-deficient SF mouse—that KO of the host CD73 gene reduced clinical symptoms, especially dermal inflammation, including ear deformity and dermatitis of the ears and tail. Also, the double KO mildly prolonged the lifespan of SF mice, even though CD73 deletion could not relieve the systemic inflammation caused by Treg deficiency.

The SF mouse mimics human IPEX syndrome, with symptoms of dermatitis and multiple organ inflammation.^17^ IPEX syndrome is a rare condition, only occurring in 1 in only 1,000,000 people; but it is beginning to gain attention in the research sphere.^3^^,^^18^^,^^19^ Importantly, studies in SF mice could facilitate the understanding of the role(s) of the Foxp3 gene in transcriptive control of other genes associated with Treg cell development, stability, and function and could lead to therapeutic strategies for the IPEX syndrome, IPEX-like syndromes, and other diseases related to Treg deficiency or dysfunction.^4^

We have previously shown that intragastric administration of the probiotic *L. reuteri* DSM 17938 reduced inflammation and increased the survival of SF mice by correcting SF gut microbial dysbiosis.^7^^,^^9^ We discovered a mechanism of DSM 17938 in reducing inflammation, characterized by probiotic-derived adenosine/inosine attributable to microbial genes that encode ecto-5′-nucleotidase, equivalent to CD73.^6–8^ Treg cells facilitate CD39/CD73 generation of adenosine from tissue-released ATP to control inflammatory T cells via the interaction of adenosine with adenosine receptor 2A expressed on inflammatory T cells. Treg-deficient SF mice lacked adenosine generated by Treg cells, which could be replaced by probiotic-derived adenosine to control inflammation. In the current study, we intended to explore the probiotic-derived adenosine effect by generating host CD73-deleted SF mice, and we expected that KO of host CD73 in SF mice would yield a severe phenotype. However, we discovered that KO of host CD73 gene resulted in a milder clinical disorder. Over 50% of mice demonstrated mild dermatitis in tails and ears, as well as thin and corrected deformed ears, almost like normal ears compared with SF mice. Histological observations showed that CD73KOSF mice had reduced tail inflammation compared with SF mice evidenced by reduced thickness of the epidermis + dermis and dramatically reduced leukocyte infiltration. In addition, more CD73KOSF mice, compared with SF mice, were able to survive beyond 30 days of life. The milder clinical phenotype of SF mice caused by KO of CD73 led to consideration of the multifaceted role of CD73 in the host.

It has been widely recognized that deviations in the CD39/CD73 mechanism affect the immune tolerance to self-antigens and gives rise to severe autoimmunity.^14^ CD39 and CD73 are both expressed on Foxp3^+^Treg cells.^3^^,^^14^ Currently there is limited information on CD39 and CD73 expressions on Foxp3^−^NonTreg cells as well as how CD73 deficiency could affect CD39 expression in Treg-deficiency syndromes. We found that among non-Treg cell populations in splenocytes and blood, there are cells expressing CD39 or CD73. Moreover, a higher % of CD39^+^T cells were identified in SF mice compared with WT controls. KO of CD73 had a minimal effect on CD39^+^T cells, except for a reduction in CD39^+^T cells in the spleen. This information suggests that CD39 and CD73 are present not only on Treg cells, but also on Foxp3^−^NonTreg cells, mainly inflammatory effector T cells.^20^ How the presence of CD39 and CD73 on inflammatory T cells could mitigate severe dermatitis needs to be further explored.

SF mice had significantly increased CD4^+^ and CD8^+^T cells compared with WT mice in the spleen and blood. KO of CD73 in SF mice normalized both cell numbers, which led us to question a potential association between KO of CD73 and thymus development. The thymus controls a constant production of self-tolerant T cells throughout life. The outer cortex is where early-stage thymocytes develop and the inner medulla is the site where later-stage thymocytes develop. The intersection of these regions is the cortical-medullary junction, where both immature and mature T cells reside and where blood vessels transport the hematopoietic progenitors from the bone marrow to the thymus. Mature T cells from the thymus then migrate to the peripheral lymphoid organs.^21^ We found that T cell numbers were reduced in the thymus of CD73KOSF mice, leading to the speculation that CD73 affects T cell development in the thymus.

Previous work showed that although few thymocytes are CD73^+,22^ CD73 is present on thymus mesenchymal stromal cells (TMSCs).^21^ TMSCs provide signals that are essential for thymocyte development. For example, TMSCs promote the homing of thymic-seeding progenitors, induce T cell differentiation, and support thymocyte proliferation and survival;^23^^,^^24^ however, little is known about the role of CD73 in thymic T cell development. CD73 could mediate lymphocyte-stromal cell interactions or condition the local microenvironment to facilitate lymphocyte development. Others have shown that KO of CD73 resulted in a decreased number of CD4^+^CD25^+^FoxP3^+^Treg cells in thymus.^25^ Interestingly, we observed a reduced number of thymus T cells in CD73KOSF mice, while these T cells shifted to a subset with higher CD3 expression (increased % of CD3^++^T cells). Among these, CD3^++^T cells emerged an increased % of single CD4^+^T cell subset and indicating that CD73 expression may not only promote T cell development (into DP CD4^+^CD8^+^T cells) but may also restrict the further differentiation and/or activation of these T cells (to single positive CD4^+^cells) in the thymus of SF mice. It has recently been reported that CD73 restricted mitochondrial capacity in CD8^+^T cells in vitro and that antigen-specific CD73-deficient CD8^+^T cells were more effective in reducing tumor burden in melanoma-bearing mice.^26^ Therefore, it will be important to further investigate the relationship between CD73 and nuclear transcription factors, such as Foxp3, which regulate the development of CD4^+^T cells in the thymus. CD73 may balance leukocyte subpopulations in the peripheral lymphoid tissues in autoimmune conditions.

We emphasize that KO of CD73 specifically reduced dermatitis in the tails and ears, while not altering systemic inflammation (plasma levels of inflammatory cytokines and histological liver and lung inflammation). Both SF and CD73KOSF mice demonstrated significant increases in plasma levels of proinflammatory cytokines including Th1-associated (IFN-γ and IL-12p70) and Th2-associated (IL-4 and IL-5) cytokines, as well as other proinflammatory cytokines (IL-2, IL-6, IL-15, IL27/p28/IL-30, and TNF-α). In addition, the IL-10 levels in SF mice and CD73KOSF mice were also significantly increased compared with WT mice. IL-10 is generally considered a key immunosuppressive cytokine produced by immune cells such as regulatory T cells,^27^ regulatory B cells,^28^ and macrophages.^29^ During Foxp3^+^Treg deficiency, IL-10 generated by other immune cells may enhance IL-2–stimulated proliferation of both CD4^+^ and CD8^+^T cells.^30^ IL-2 has been proposed to be a potential master regulator for autoimmunity associated with skin and lung inflammation.^31^ However, in our study, CD73KOSF mice with mild skin symptoms failed to show reduced plasma IL-2 levels compared with SF mice with severe dermatitis.

Multiorgan inflammation in SF mice likely involves not only T cells, but also monocytes/macrophages and neutrophils. MIP-1α (CCL3) is a chemoattractant for most immune cells (eg, monocytes/macrophages, CD8^+^T cells, B cells, basophils, eosinophils, mast cells and natural killer cells).^32^ MIP-2 (CXCL2) is a potent chemoattractant for neutrophils^33^ whereas MCP-1 (CCL2) regulates migration and infiltration of monocytes/macrophages.^34^ IP-10 (CXCL10) can be secreted by leukocytes, neutrophils, eosinophils, endothelial cells, fibroblastic cells, and keratinocytes in response to IFN-γ. IP-10 binds to CXCR3, which is typically expressed on activated T cells, and plays a major role in the inflammatory response.^35^ Interestingly, plasma levels of IL-33 were significantly increased in CD73KOSF mice but not in SF mice. IL-33 is a member of the IL-1 family and plays an ambivalent role in autoimmune diseases.^36^ The biological functions of IL-33 largely depend on its ligation to the corresponding receptor and ST2 (suppression of tumorigenicity 2).^37^ The IL-33/ST2 signaling has effects on both monocyte/macrophage differentiation^38^ and T cells, including Th1, Th2, and Th17 cell responses.^39^ The administration of IL-33 drives immune tolerance by increasing the de novo differentiation of Foxp3^+^Treg cells in skin-transplanted mice.^40^ A recent study showed that IL-33 induces Treg migration into barrier-disrupted skin, increasing the numbers of IL-10^+^ and TGF-β^+^Treg cells in the skin barrier disrupted area.^41^ It is likely that the IL-33 level in CD73KOSF may be associated with reduced autoimmune dermatitis.

A limitation of this current study is that it is lacking a comprehensive approach to mechanisms into how the absence of CD73 in all cells may be altering the ear and tail lesions in scurfy mice. The CD73 gene is broadly expressed in >20 organs in mice, including immune cells, lungs, intestine, brain, liver, and bladder (National Center for Biotechnology Information Gene ID: 23959). Besides reductions of central and peripheral lymphoproliferation due to the knockout of host CD73, there are several possible mechanisms related to CD73 in skin inflammation that require further investigation: (i) CD73 and skin dendritic cells (DCs), as a recent study showed that CD73 expression slows down the migration of skin DCs,^42^ implicating that KO of host CD73 prevents skin lesion in SF mice probably is associated with an enhanced migration of skin DCs in SF mice; (ii) CD73 and Foxp3, as the interactions between these 2 genes and/or downstream signaling affecting skin inflammation requires further study; (iii) IL-33, as the therapeutic effects of administering IL-33 in this model are unknown; and (iv) CD73 and other types of T cells, as γδT cells are key in the pathogenesis of skin inflammation,^43^ while importantly, CD73 is expressed on more than 90% of peripheral γδT cells in mice.^44^ In addition, tissue-specific CD8^+^T cells play an important role in skin inflammation.^45^ In the case of tumors, investigators showed that CD73 presents distinct roles in hematopoietic cells and nonhematopoietic cells to promote tumor growth.^46^ CD73 on hematopoietic cells (mainly Treg cells) suppresses systemic antitumor T cell expansion and effector function, whereas CD73 derived from nonhematopoietic cells (for example, endothelial cells) primarily affects local antitumor T cell immunity by limiting T cell (specifically CD8^+^T cell) homing to tumors. This distinct function of CD73 may imply skin tissue specifically during skin inflammation. It is likely that knockdown CD73 affects tissue-specific CD8^+^T or γδT cell infiltration to reduce skin inflammation in SF mice.

In conclusion, KO of the CD73 gene reduces dermatitis in Foxp3^+^Treg-deficient SF mice, which may mainly be due to reduced central and peripheral lymphoproliferation. However, CD73 may have a multifaceted role in regulation of inflammatory conditions. We suggest CD73 is a potential therapeutic target for improving T cell–mediated dermatitis—one of the most common symptoms in Treg deficiency–associated primary immune deficiency diseases, such as IPEX syndrome, IPEX-like syndrome, and other autoimmune diseases related to Treg dysfunction.^4^

## Data Availability

The data that support the findings of this study are included in the article. Further inquiries can be directed to the corresponding author.
